# Near-Infrared Fluorescence Imaging of Mammalian Cells and Xenograft Tumors with SNAP-Tag

**DOI:** 10.1371/journal.pone.0034003

**Published:** 2012-03-30

**Authors:** Haibiao Gong, Joy L. Kovar, Brenda Baker, Aihua Zhang, Lael Cheung, Daniel R. Draney, Ivan R. Corrêa, Ming-Qun Xu, D. Michael Olive

**Affiliations:** 1 LI-COR Biosciences, Lincoln, Nebraska, United States of America; 2 New England Biolabs, Ipswich, Massachusetts, United States of America; University of Texas, M.D. Anderson Cancer Center, United States of America

## Abstract

Fluorescence in the near-infrared (NIR) spectral region is suitable for *in vivo* imaging due to its reduced background and high penetration capability compared to visible fluorescence. SNAP_f_ is a fast-labeling variant of SNAP-tag that reacts with a fluorescent dye-conjugated benzylguanine (BG) substrate, leading to covalent attachment of the fluorescent dye to the SNAP_f_. This property makes SNAP_f_ a valuable tool for fluorescence imaging. The NIR fluorescent substrate BG-800, a conjugate between BG and IRDye 800CW, was synthesized and characterized in this study. HEK293, MDA-MB-231 and SK-OV-3 cells stably expressing SNAP_f_-Beta-2 adrenergic receptor (SNAP_f_-ADRβ2) fusion protein were created. The ADRβ2 portion of the protein directs the localization of the protein to the cell membrane. The expression of SNAP_f_-ADRβ2 in the stable cell lines was confirmed by the reaction between BG-800 substrate and cell lysates. Microscopic examination confirmed that SNAP_f_-ADRβ2 was localized on the cell membrane. The signal intensity of the labeled cells was dependent on the BG-800 concentration. *In vivo* imaging study showed that BG-800 could be used to visualize xenograph tumors expressing SNAP_f_-ADRβ2. However, the background signal was relatively high, which may be a reflection of non-specific accumulation of BG-800 in the skin. To address the background issue, quenched substrates that only fluoresce upon reaction with SNAP-tag were synthesized and characterized. Although the fluorescence was successfully quenched, *in vivo* imaging with the quenched substrate CBG-800-PEG-QC1 failed to visualize the SNAP_f_-ADRβ2 expressing tumor, possibly due to the reduced reaction rate. Further improvement is needed to apply this system for *in vivo* imaging.

## Introduction

Fluorescence has been extensively used in biological research to visualize molecular and cellular events. Its application ranges from visualizing targeting molecules in single cells to imaging physiological and pathological alterations in whole animals [1], [2]. Its high sensitivity and stability, and simplicity of multiplexing offer advantages over other imaging methods in many applications. The most commonly used fluorophores include organic dyes, fluorescent proteins and quantum dots [1]. Each class of fluorophores has its own advantages and limitations. For example, fluorescent proteins can be easily expressed in cells and whole organisms. On the other hand, fluorescent organic dyes are more suitable for conjugation to other molecules, such as nucleic acids and proteins.

It is of great interest to develop fluorophores with excitation (Ex) and emission (Em) maxima in the near-infrared (NIR) region (700–900 nm). With fluorescence in the NIR region, cells, buffers and plastic materials used in assays have reduced background. As a result, NIR fluorescence imaging offers higher sensitivity and better signal-to-background (S/B) ratio compared to visible spectra. More importantly, due to the reduced light absorption and scattering of NIR light in animal tissues, and the low tissue autofluorescence in the NIR region, NIR fluorescence is well-suited for *in vivo* animal imaging [2], [3], [4]. Significant efforts have been made to shift the spectra of the fluorescent proteins to longer wavelengths [5], [6], [7], [8]. The most red-shifted fluorescent proteins are bacteriophytochrome-based near-infrared fluorescent proteins IFP1.4 [8] and iRFP [9]. However, the Ex/Em peaks of IFP1.4 (Ex/Em: 684/708 nm) and iRFP (Ex/Em: 690/713 nm) are still significantly lower compared to those of NIR fluorescent dyes such as IRDye 800CW (Ex/Em: 774/789 nm).

SNAP_f_ is a fast-labeling variant of SNAP-tag, which is derived from the human DNA repair protein O^6^-alkylguanine-DNA-alkyltransferase (AGT) [10]. It reacts specifically and rapidly with benzylguanine (BG) derivatives, leading to covalent labeling of the SNAP_f_ with a variety of functional moieties, such as fluorescent dyes, biotin and solid surfaces. The fusion of SNAP_f_ to a protein of interest yields a tagged protein capable of forming a covalent linkage to fluorescent dyes [11], [12].

The NIR fluorescent dye IRDye 800CW has been conjugated to a variety of molecules for different applications. Examples include labeled antibodies for Western, In-Cell-Western, and labeled 2-deoxyglucose, RGD peptide and target-specific peptides for animal imaging [13], [14], [15]. An epidermal growth factor receptor (EGFR)-specific Affibody molecule labeled with IRDye 800CW has been successfully used in cell-based plate assays, microscopic examination, live animal and tissue section imaging studies [15]. Recently, a toxicity study on IRDye 800CW revealed that there was no observed adverse effect at a dose of approximately 10,000 times higher than the projected dose for *in vivo* imaging. This is the first toxicity study on a NIR dye with the functional labeling potential [16].

In this study, the BG-800 substrate was synthesized by a one-step reaction between IRDye 800CW-NHS ester and BG-NH2. BG-800 was characterized using both cell-based assay and *in vivo* imaging. To reduce the background, quenched substrates containing IRDye 800CW and IRDye QC1 conjugated at the benzyl and guanine groups of BG, respectively, were created and characterized.

## Materials and Methods

### Ethics statement

All experimental procedures for the use of animals were previously reviewed and approved by the institutional animal care and use committee (IACUC) at the University of Nebraska-Lincoln (protocol #402), and all of the experiments were conducted in accordance with the Guide for the Care and Use of Laboratory Animals published by the US National Institutes of Health.

### Chemicals and reagents

The SNAP_f_-Beta-2 adrenergic receptor (SNAP_f_-ADRβ2) vector, amine-terminated building block BG-NH2, BG-782 (SNAP-Surface 782) and purified SNAP_f_-EGF protein were from New England Biolabs (Ipswich, MA). The IRDye 800CW and IRDye QC1 were from LI-COR Biosciences (Lincoln, NE). The synthesis of BG substrates (BG-800, CBG-800-QC1 and CBG-800-PEG-QC1) was conducted at New England Biolabs based on previously published methods [10]. The structures of these substrates are shown in Fig. S1. All substrates were reconstituted in DMSO to 1 mM as stock solutions. TO-PRO-3 and DAPI nucleus staining reagents were purchased from Invitrogen (Carlsbad, CA).

### Cell culture

The human ovarian adenocarcinoma cell line SK-OV-3, breast adenocarcinoma cell line MDA-MB-231 and embryonic kidney 293 (HEK293) cells were obtained from the American Type Culture Collection (ATCC, Manassas, VA). HEK293 and MDA-MB-231 cells were maintained in Dulbecco's Modified Eagle's Medium (DMEM) supplemented with 10% fetal calf serum (FBS) and 1% penicillin-streptomycin (complete DMEM). SK-OV-3 cells were maintained in McCoy's 5 A medium (McCoy) supplemented with 10% fetal calf serum (FBS) and 1% penicillin-streptomycin (complete McCoy).

To collect cell lysates, cells were rinsed with PBS once before adding RIPA buffer. The cells were then incubated on ice in RIPA buffer for 15 min. The cell lysates were collected and centrifuged at 4°C to separate the supernatant from the cell debris.

### Cell transfection and stable cell line generation

The cell transfection procedure was modified from the previously described method [17]. Lipofectamine 2000 (Invitrogen, Carlsbad, CA) was used to deliver the SNAP_f_-ADRβ2 plasmid into the cells. Two days after transfection the cells could be used for *in vitro* imaging studies as described below. For stable cell selection, the culture medium was replaced with that contains G418 at 24 h after transfection. The G418 resistant cells were pooled and stored for future use.

### Reaction of BG substrates with cell lysate

BG substrates were added to cell lysate (1 µg/µl) to a final concentration of 500 nM. The reaction was conducted at room temperature. The reaction mixtures were resolved by gel electrophoresis. The labeled protein bands were visualized by scanning the gel on an Odyssey Infrared Imaging System (LI-COR Biosciences).

### Cell staining with BG substrates in 96-well plates

Cells were seeded at approximately 3×10^4^ (MDA-MB-231), 2×10^4^ (HEK293) or 1×10^4^ (SK-OV-3) cells per well in 96-well plates and cultured overnight before the assay. The cell density was about 80–90% confluent at the time of assay. The BG substrates were diluted in complete cell culture medium to designated concentrations and incubated in the 37°C 5% CO_2_ incubator for 30 min except where otherwise stated. Cells were fixed with 3.7% formaldehyde/PBS and washed with PBS containing 0.1% Tween 20 (PBST), and then incubated in TO-PRO-3 nucleus stain agent (1∶5000 in PBS) to normalize for cell numbers. After three additional washes with PBST, the plates were scanned and signal intensity quantified [18].

### Microscopic analysis

Cells were seeded in 6-well plate with cover slips and cultured overnight. The cells were incubated with 1 µM BG substrates at 37°C 5% CO_2_ for 30 min, fixed, permeabilized and washed as described above. Instead of TO-PRO-3, DAPI nucleus staining agent was used to visualize the nuclei by microscopy. After the final wash, the cover slips were mounted on glass slides with Fluoromount reagent (Sigma, St. Louis, MO). The images were acquired using an Olympus IX81 Inverted microscope system equipped with a halogen bulb (Olympus, Hamburg, Germany). NIR filters (EX: 710/75 nm, EM: 810/90 nm; Chroma Technology Corp., Rockingham, VT) were used for IRDye 800CW detection. The images were deconvolved using the accompanying software.

### Xenograft mouse model

Mice were maintained on a purified maintenance diet (AIN-93M) from Harlan Teklad (Madison, WI). The xenograft tumors were established as previously described with modifications [19]. In brief, athymic nude (nu/nu) mice, obtained from Charles River Laboratories, Inc. (Cambridge, MA) at 4 weeks of age, were subcutaneously injected with 5×10^6^ SK-OV-3 cell suspension in 0.1 ml serum-free media. Imaging studies began when tumors reached about 5 mm in size.

### *In vivo* animal imaging

Mice were anesthetized with 2% isoflurane throughout the procedures. For imaging experiments, the BG substrates were diluted in 100 µl PBS and injected through the tail vein. The images were acquired at indicated time points with a Pearl Impulse Imager (LI-COR Biosciences). The Ex/Em settings for the 700 nm channel and 800 nm channel were 685/720 nm and 785/820 nm, respectively. The images were analyzed using the accompanying software [14].

### Organ and tissue analysis

Mice were sacrificed at 1 d after BG substrates injection, and dissected to collect the organs. The excised organs were rinsed in PBS, and imaged using a Pearl Impulse Imager. For gel analysis, tissue samples were homogenized in RIPA buffer. After centrifugation, the supernatants were run on a SDS-PAGE gel and analyzed by in-gel fluorescence scanning.

## Results

### BG-800 labeling of cells transiently transfected with SNAP_f_-ADRβ2

**Figure 1:**
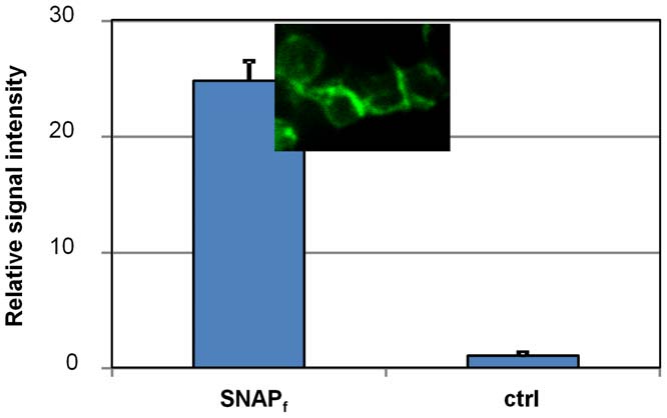
Reaction of BG-800 with HEK293 cells expressing SNAP_f_-ADRβ2 protein. HEK293 was transiently transfected with SNAP_f_-ADRβ2 expression plasmid. After 2 d of culture, cells were reacted with BG-800. The fluorescence signal on the cells was measured by scanning on Odyssey Infrared Imaging System. HEK293 cells transfected with empty vector were used as a control. Shown in the inset is a representative microscopic image of BG-800 stained HEK293 cells expressing SNAP_f_-ADRβ2.

A SNAP_f_-ADRβ2 expressing plasmid was used in this study. The ADRβ2 portion of the fusion protein directs the localization of the protein to the cell membrane. HEK293 cells were transiently transfected with SNAP_f_-ADRβ2 using a Lipofectamine 2000-mediated method. After 2 d culture, the transfected cells were labeled with BG-800. Because the labeling is irreversible, excess substrate could be washed away [12]. The fluorescence signal on SNAP_f_-ADRβ2 transfected cells was about 24 times higher than that of un-transfected HEK293 cells (Fig. 1), indicating that the BG-800 substrate could react with SNAP_f_-ADRβ2 protein. Microscopic examination revealed that the fluorescence signal was mainly on the cell membrane (Fig. 1, inset).

### Generation of stable cells expressing SNAP_f_-ADRβ2 and labeling with BG-800

**Figure 2:**
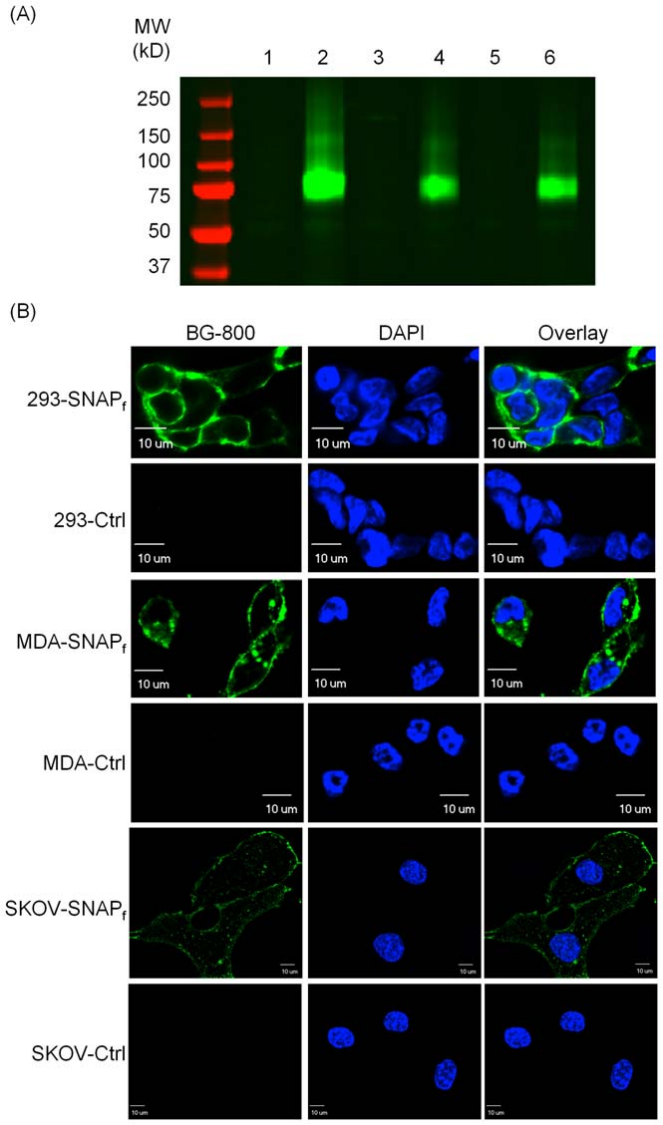
Confirmation of SNAP_f_-ADRβ2 stable cell lines. (A) HEK293, MDA-MB-231, and SK-OV-3 cells stably expressing SNAP_f_-ADRβ2 (designated as 293-SNAP_f_, MDA-SNAP_f_ and SKOV-SNAP_f_, respectively) were lysed in RIPA buffer. The cell lysates were reacted with BG-800 substrate and run on a SDS-PAGE gel. The 70-kDa bands represented the SNAP_f_-ADRβ2 protein. The parental cell lines of the stable cells were used as negative controls. 1, HEK293; 2, 293-SNAP_f_; 3, MDA-MB-231; 4, MDA-SNAP_f_; 5, SK-OV-3; 6, SKOV-SNAP_f_. (B) Microscopic examination of BG-800 reaction with stable cell lines expressing SNAP_f_-ADRβ2. 293-SNAP_f_, MDA-SNAP_f_ and SKOV-SNAP_f_ cells were incubated with BG-800 in complete culture medium. The cells were then fixed and stained with DAPI to show the nuclei. The parental cells of each cell lines were used as negative controls. Note that BG-800 reaction signals were mainly on the cell membrane. Scale bar: 10 µm.

HEK293, MDA-MB-231 and SK-OV-3 cells stably expressing SNAP_f_-ADRβ2 were selected using G418-containing medium. These cells were designated as 293-SNAP_f_, MDA-SNAP_f_ and SKOV-SNAP_f_, respectively. The expression of SNAP_f_-ADRβ2 in the stable cells was determined by the reaction between BG-800 and cell lysates. Fig. 2A shows a representative gel image in which the reaction mixtures were resolved. Each of the three stable cell lines showed a positive band, presumably resulting from the reaction between SNAP_f_-ADRβ2 and BG-800. As a comparison, this band was absent for parental cell lines. The calculated molecular weight of SNAP_f_-ADRβ2 is about 70-kDa, which matches the molecular weight of the bands on the gel.

Microscopic examination also confirmed the SNAP_f_-ADRβ2 expression in the stable cells. It also demonstrated that the fluorescence signal was predominantly on the cell membrane (Fig. 2B), indicating the correct localization of SNAP_f_-ADRβ2 protein. However, signals were also observed inside the cells, probably representing internalized SNAP_f_-ADRβ2 after labeling.

### The reaction signal is dependent on BG-800 concentration

**Figure 3:**
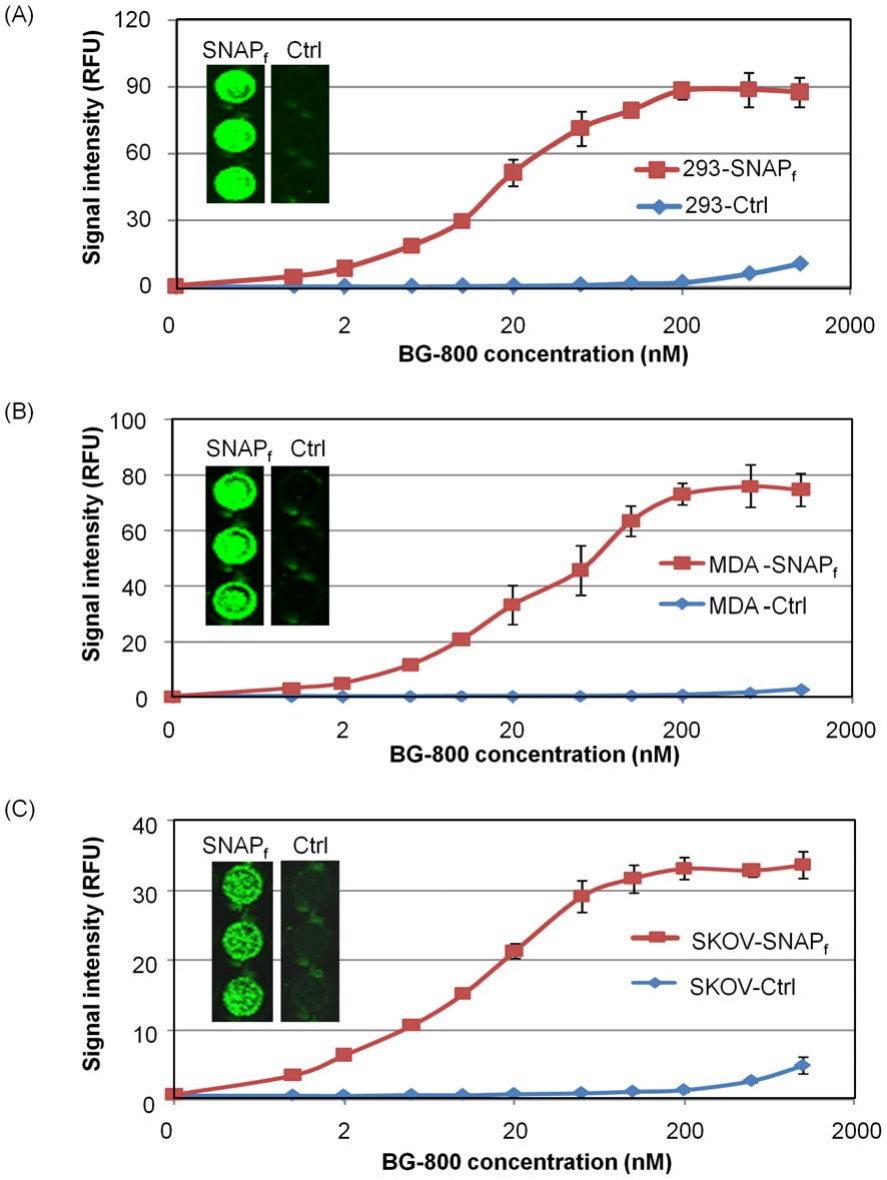
Concentration dependent reaction of BG-800 with stable cells. 293-SNAP_f_ (A), MDA-SNAP_f_ (B) and SKOV-SNAP_f_ (C) stable cells were reacted with different concentrations of BG-800. The fluorescence signals from the reaction were scanned on Odyssey Infrared Imaging System and quantified. TO-PRO-3 staining was used as the internal control. The insets represent scanned images of BG-800 (100 nM) stained stable cells (SNAP_f_) and control cells (Ctrl). RFU, relative fluorescence unit.

The reaction signals between BG-800 and SNAP_f_-ADRβ2 stable cells were dependent on BG-800 concentration. With the increase of BG-800 concentration, the signal intensity also increased (Fig. 3). All three cell lines showed a similar trend, and the maximum signals were reached when approximately 200 nM BG-800 was applied. As a comparison, the parental cell lines were reacted with the same concentrations of BG-800, and minimal signals were observed (Fig. 3A–C, and inset), lending additional support that the signal from stable cells is specific.

### The combination of BG-800 with SNAP_f_-ADRβ2 offers better signal to background ratio than other systems

**Figure 4:**
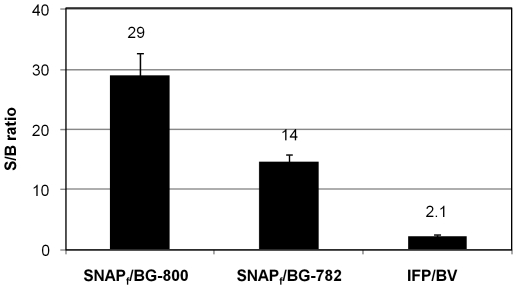
Comparison of signal to background (S/B) ratios of different systems. HEK293 cells expressing either SNAP_f_-ADRβ2 or IFP1.4 were used. Both SNAP_f_-ADRβ2 and IFP1.4 are under the control of the CMV promoter. SNAP_f_-ADRβ2 expressing cells (293-SNAP_f_) were stained with BG-800 (SNAP_f_/BG-800) or BG-782 (SNAP_f_-BG-782), respectively. IFP1.4 expressing cells were incubated with biliverdin (IFP/BV). The excess substrate and biliverdin were washed away after reaction. The fluorescence signals of the cells were scanned on the culture plate without trypsinization and concentration. Signals were detected at their respective optimal wavelength (Ex/Em: 785/820 nm for BG-800 and BG-782; 685/720 nm for IFP1.4). The signals of HEK293 parental cells stained with the respective substrates or biliverdin were defined as background.

To compare BG-800 with the commercially available SNAP-tag substrate BG-782, 293-SNAP_f_ and its parental cell line HEK293 were labeled with either BG-800 or BG-782. The signal intensity of BG-800 reaction with 293-SNAP_f_ was approximately five-fold higher than that of BG-782 (data not shown). The ratio of 293-SNAP_f_ signal to HEK293 signal is defined as the signal-to-background (S/B) ratio. The S/B ratio of BG-800 (S/B = 29) was about two-fold higher than that of BG-782 (S/B = 14) (Fig. 4). We also generated HEK293 cells stably expressing the NIR fluorescent protein IFP1.4 [8]. The resulting stable cell line 293-IFP was also compared with 293-SNAP_f_/BG-800. The ratio of 293-IFP signal to HEK293 signal after biliverdin treatment was 2.1, which is about 14 times lower than that of 293-SNAP_f_/BG-800.

### Tumors expressing SNAP_f_-ADRβ2 can be visualized by BG-800

**Figure 5:**
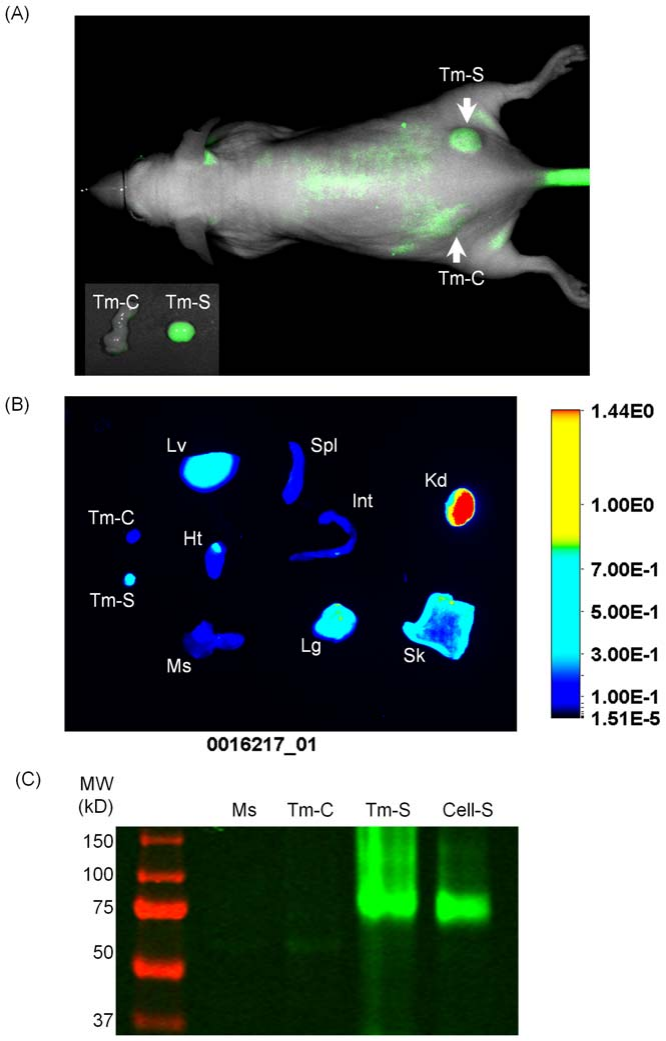
*In vivo* imaging with BG-800 substrate. (A) Xenograft tumors were established using either SK-OV-3 parental cells as the control (Tm-C, left side) or SKOV-SNAP_f_ stable cells (Tm-S, right side). The mice were imaged 24 h after i.v. injection of 10 nmol of BG-800 substrate. The tumors were indicated with arrows. The tumors were dissected and imaged *ex vivo* after whole animal imaging (lower left corner inset). (B) Tissue distribution of BG-800. Tissues were dissected 24 h after BG-800 administration and imaged. Note that the tissues were from a different mouse as shown in (A). Ht, Heart; Int, intestine; Kd, kidney; Lg, lung; Lv, liver; Ms, muscle; Sk, skin; Spl, spleen; Tm-C, SK-OV-3 tumor; Tm-S, SKOV-SNAP_f_ tumor. (C) Gel analysis of tissue lysates from Ms, Tm-C, Tm-S and SKOV-SNAP_f_ cell lysate reacted with BG-800 (Cell-S).

The BG-800 substrate was evaluated *in vivo* in mouse models. Nude mice bearing SKOV-SNAP_f_ tumors (Tm-S) on one side and SK-OV-3 tumors (Tm-C) on the other side were injected with 10 nmol of BG-800 through the tail vein. Whole mouse images were acquired at 24 h after imaging agent administration. Fig. 5A showed that SNAP_f_-ADRβ2 expressing tumor could be visualized by BG-800 at 24 h post injection. The higher fluorescence signal in Tm-S was revealed more clearly by *ex vivo* imaging after tissue dissection (Fig. 5A, inset). The ratios of Tm-S/Tm-C and Tm-S/muscle were 3.31±0.43 and 12.3±2.9, respectively. However, it was noticed that the background fluorescence signal was high. This high background signal might be caused by accumulation of BG-800 in the skin, as demonstrated by *ex vivo* imaging analysis (Fig. 5B). Other organs with high BG-800 accumulation included kidney, liver and lung.

To assess whether the tumor signal was from the specific labeling of SNAP_f_-ADRβ2 by BG-800, the tumor lysate was analyzed by gel electrophoresis. Fig. 5C showed that Tm-S lysate contained a product with the same size as the product from the reaction between BG-800 and SKOV-SNAP_f_ cell lysate. In comparison, neither muscle nor SK-OV-3 tumor (Tm-C) contained this product. This product was also absent from other tissue lysates, including liver, lung and kidney (data not shown).

### Quenched BG substrates

To assess whether the non-specific background signal could be reduced by using quenched substrates, CBG-800-QC1 was synthesized by conjugating IRDye 800CW and IRDye QC1to the benzyl and guanine groups, respectively. To decrease the adverse steric effect of the bulky IRDye QC1, a PEG linker was incorporated between IRDye QC1 and guanine. This version of quenched substrate with a PEG linker was designated as CBG-800-PEG-QC1 (Fig. S1). The quenching efficiencies of CBG-800-QC1 and CBG-800-PEG-QC1 were estimated to be 97% and 94%, respectively.

**Figure 6:**
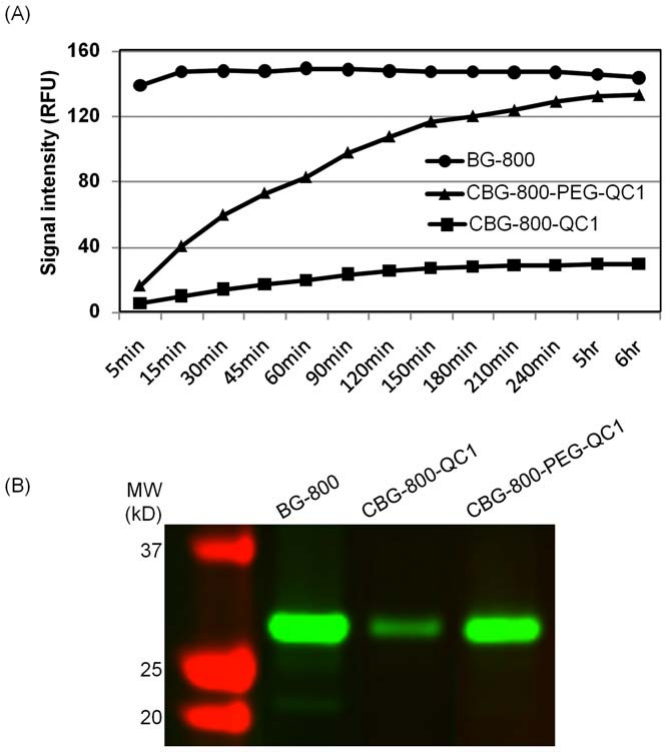
Reaction of BG-800, CBG-800-QC1 and CBG-800-PEG-QC1 substrates with purified SNAP_f_-EGF protein. (A) BG substrates (100 nM) were incubated with SNAP_f_-EGF protein (15 ng/µl) in a 96-well plate. The plate was scanned after different time periods of reaction to measure the fluorescence signal. (B) The reaction mixtures of SNAP_f_-EGF with different BG substrates were resolved on a SDS-PAGE gel and scanned to visualize the labeled protein.

When incubated with excess purified SNAP_f_-EGF protein, the reaction signal from CBG-800-PEG-QC1 increased over time, and approached the level of BG-800 after 6 h (Note that unreacted BG-800 was not separated from the reaction mixture). In contrast the reaction signal from CBG-800-QC1 was only 21% of that of BG-800 after the same period of reaction (Fig. 6A). Analysis by gel electrophoresis demonstrated that although the intensities varied, the molecular weights of the reaction products from all BG substrates were the same (Fig. 6B). Reaction with SNAP_f_-ADRβ2 expressing cells revealed that CBG-800-PEG-QC1 produced stronger signal than CBG-800-QC1. However signals from both CBG-800-PEG-QC1 and CBG-800-QC1 were much weaker compared to that of BG-800 (data not shown), indicating that a quencher on the guanine adversely affected the reactivity.

As the reaction performance of CBG-800-PEG-QC1 was superior compared to BG-800- QC1, CBG-800-PEG-QC1 was used for *in vivo* animal imaging tests. Minimal background signal was observed for the quenched substrate even at the early stage post probe injection when the clearance had not occurred (Fig. S2), indicating that the quenching effect of the probe persevered *in vivo*. However, CBG-800-PEG-QC1 failed to detect the SNAP_f_-ADRβ2 expressing tumors under these conditions.

## Discussion

Fluorescence technology has become an indispensable tool for biological and biomedical research. SNAP-tag fluorescence imaging has been used in various applications, including protein-protein interaction [20], hydrogen peroxide detection [21], monitoring zinc flux [22], virus-cell interactions [23] and super-resolution imaging of live cells [24]. SNAP_f_ used in this study is a fast-labeling variant of SNAP-tag with some extra mutations [10]. As a new technology discovered less than a decade ago [12], the SNAP-tag has some advantages over fluorescent proteins. Firstly, NIR SNAP-tag substrates could be synthesized readily from NIR dyes. As a result, NIR fluorescence imaging is greatly facilitated with SNAP-tag. This is in contrast to the efforts needed to engineer NIR fluorescent proteins. Secondly, a variety of fluorescent substrates with different colors could be used to label one single SNAP-tag without a requirement for separate cloning and expression for each color. Once a stable cell line or transgenic animal is established, the color on the SNAP-tag can be easily altered by using a substrate with different Ex/Em spectra. However, changing to a different color with fluorescent proteins entails laborious processes of re-establishing stable cell lines or transgenic animals *de novo*. Thirdly, the labeling time with SNAP-tag can be controlled easily, allowing for “pulse-chase” experiments that require labeling with different probes at different time points.

Although SNAP-tag technology has been widely used in cell imaging, much less work has been done to apply this technology in animal imaging. A fusion protein of SNAP-tag with a single-chain antibody fragment has enabled targeting EGFR-overexpressing tumors. However, the labeling of SNAP-tag by NIR substrates was conducted *in vitro* in that study [25]. In a recent report, BG-782 was successfully used to label SNAP-tag *in vivo*. Tumors expressing SNAP-tag fusion proteins were visualized and the half-lives of SNAP-tag fusion proteins were measured *in vivo* [26].

A NIR fluorescent SNAP-tag substrate BG-800 was synthesized by conjugating IRDye 800CW to BG-NH2. Because BG-800 is cell impermeable, we choose to use the SNAP_f_-ADRβ2 fusion protein, in which ADRβ2 directs the localization of SNAP_f_ fusion protein to the cell membrane. BG-800 reacted with SNAP_f_-ADRβ2 in both cell lysate and live cell culture. It was also found that BG-800 produced a higher signal and S/B ratio compared to BG-782 in cell-based assay. The tumors expressing SNAP_f_-ADRβ2 could be visualized by BG-800. The signal was from the specific reaction between SNAP_f_-ADRβ2 and BG-800, as evidenced by gel analysis of protein exacts from the dissected tumors. However the relatively high signal in the skin, presumably due to the non-specific accumulation of BG-800 in this tissue, produced high background. The accumulation of BG-800 in the skin is unlikely caused by IRDye 800CW because little skin signal has been detected with either free dye or various other IRDye 800CW conjugates, including small organic molecules (such as 2-DG), small peptides (such as RGD), large peptides (such as EGF, Affibody) and antibodies [14], [15], [27], [28]. It is also noteworthy that a HaloTag probe containing IRDye 800CW was used to detect HaloTag expressing tumors. No skin accumulation problem was noted in that study [29].

One possible solution to the background issue is to use quenched substrates, which only fluoresce upon reaction with SNAP-tag. The quenched substrates are desirable in cell-based assay because they could eliminate the wash step which is needed for the conventional unquenched substrates [10], [30]. More importantly, the quenched substrate will produce minimal background when used in animal imaging, where the clearance of the substrate from the body is more difficult and much slower compared to the cell culture system. Various quenching mechanisms, such as self-quenching, Förster resonance energy transfer (FRET), H-dimer formation and photon-induced electron transfer (PeT), have been employed in fluorescence imaging [31], [32]. As guanine is known to quench the fluorescence of certain dyes by PeT, various dyes were tested for their quenching efficiency by guanine after conjugation to BG. Several BG substrates were discovered to have a strong (>10-fold) increase in their fluorescence upon covalent labeling of the SNAP-tag [33]. A more general method is based on the FRET principle. A fluorescent dye and a quencher could be linked to the benzyl moiety and the guanine moiety, respectively. In this substrate, the fluorescence of the dye is quenched by the closely-linked quencher. After reaction with SNAP-tag, the guanine-quencher group will separate from the benzyl-dye group, resulting in the restoration of the fluorescence. The quencher was linked to the C-8 or N-9 positions, and the resulting substrates were characterized. Although the substrates were quenched in both situations, the C-8 modification exhibited better reaction kinetics [30], [34]. A variety of quenched substrates with different combinations of fluorescent dyes and quenchers linked at C-8 position have been synthesized and tested [10]. However, none of these quenched substrates has Ex/Em spectra in the NIR region.

We synthesized the NIR quenched substrate CBG-800-QC1 by conjugating IRDye 800CW and IRDye QC1 at the benzyl group and C-8 position of guanine group, respectively. The reaction speed of this substrate was greatly reduced compared to the unquenched BG-800. This is not surprising given that C-8 modification has been shown to adversely affect the reaction rate of the substrate [30]. It has also been reported that different quenchers affect the binding and conjugation of the substrate to the SNAP_f_ differently [10]. IRDye QC1 (MW 1244) is a relatively large molecule [35], and may hinder the access of the substrate to the active site of the enzyme. A PEG linker between IRDye QC1 and guanine improved the reaction rate, possibly by alleviating the hindrance effect of IRDye QC1. However this PEG containing quenched substrate CBG-800-PEG-QC1 failed to visualize the SNAP_f_-ADRβ2 expressing tumors. Higher doses up to three times of that used for BG-800 were tried without any significant improvement (Gong et al., unpublished data). These results indicate that the reaction rate of CBG-800-PEG-QC1 is not fast enough to match the quick body clearance of the substrate in the system we used. Although it is possible that CBG-800-PEG-QC1 may be used to visualize tumors established from other cell lines with higher SNAP_f_-ADRβ2 expression levels, or tumors expressing different SNAP-tag fusion proteins, our results suggest that improvement is needed to make this system suitable for general imaging applications. Several strategies could be envisioned to achieve this goal. Firstly, it might be beneficial to replace the bulky QC1 by smaller quenchers such as BHQ-3, which could also quench emission of NIR fluorescent dyes [36]. Secondly, a SNAP-tag mutant could be selected specifically for the quenched substrates. Thirdly, a quenched substrate with a longer circulation time *in vivo* could also improve the performance.

Fluorescence optical imaging has the advantage of multiple channels, which can be employed to image two or more targets simultaneously [37], [38]. A second version of AGT-based tag named CLIP-tag, which reacts specifically with benzylcytosine (BC) derivatives, has also been developed [39]. Because SNAP-tag and CLIP-tag only react with their specific substrates, they could be used simultaneously for dual-color fluorescence imaging. The SNAP-tag can also be combined with other protein-tags, such as HaloTag [29], or other reporter gene systems that use fluorescent substrates, such as β-galactosidase/DDAOG system [40], to create multiplexed imaging systems.

## Supporting Information

Figure S1 **Structures of BG substrates.** (A) BG-800. (B) CBG-800-QC1. (C) CBG-800-PEG-QC1.
(TIF)

Figure S2 **Comparison of BG-800 and CBG-800-PEG-QC1** ***in vivo*** **.** Nude mice were injected with 10 nmol BG-800 or CBG-800-PEG-QC1 and imaged at different time points. Note that the fluorescence signal of CBG-800-PEG-QC1-injected mouse was much lower than that of BG-800-injected mouse.
(TIF)
