# Supplementary figures and images for: Near-Infrared Fluorescence Imaging of Mammalian Cells and Xenograft Tumors with SNAP-Tag
Source: PLoS One. 2012 Mar 30;7(3):e34003. doi: 10.1371/journal.pone.0034003 (PMC3316518; doi:10.1371/journal.pone.0034003)

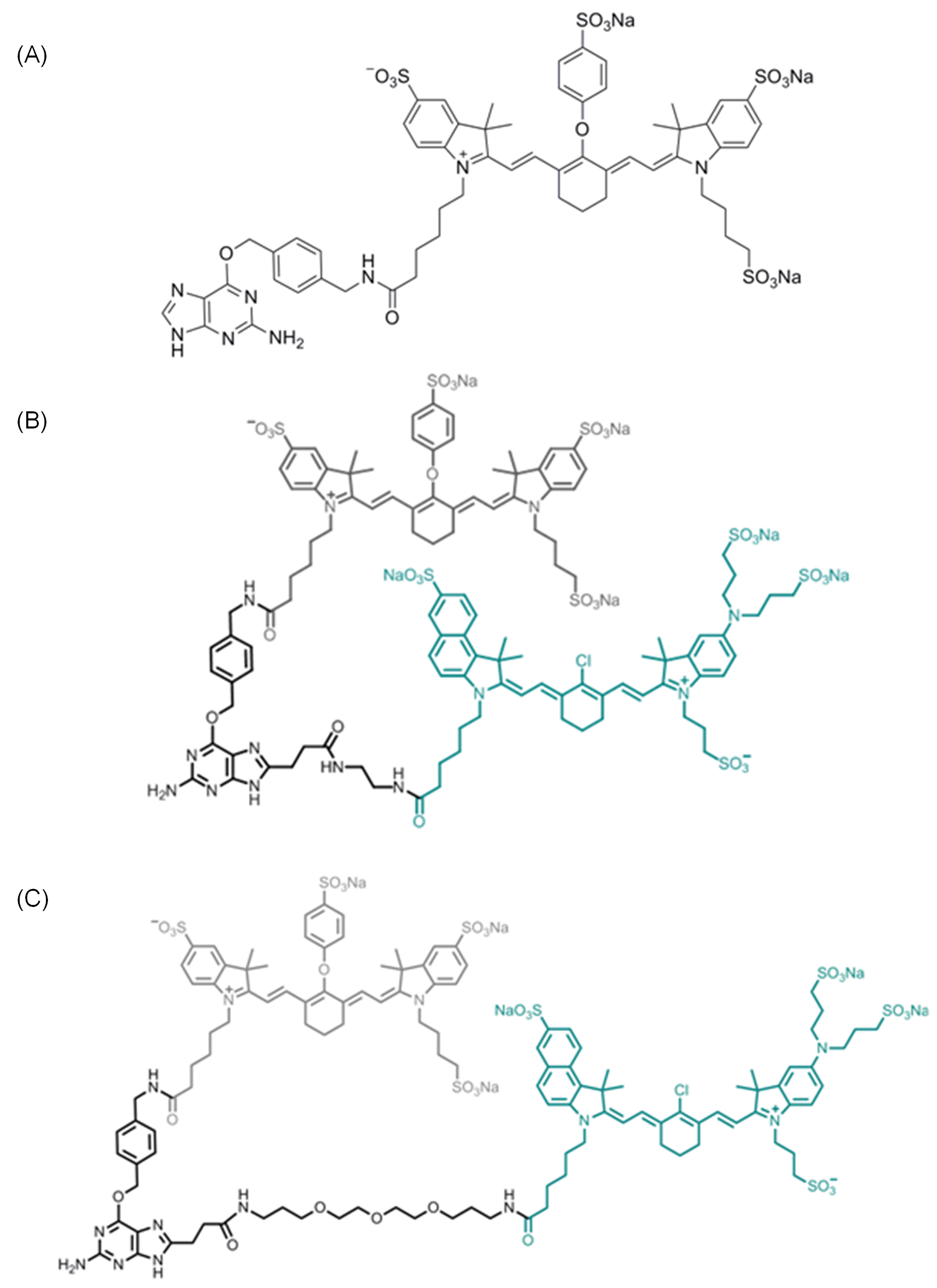

Supplement: Figure S1 — Structures of BG substrates. (A) BG-800. (B) CBG-800-QC1. (C) CBG-800-PEG-QC1. (TIF) [file pone.0034003.s001.tif]

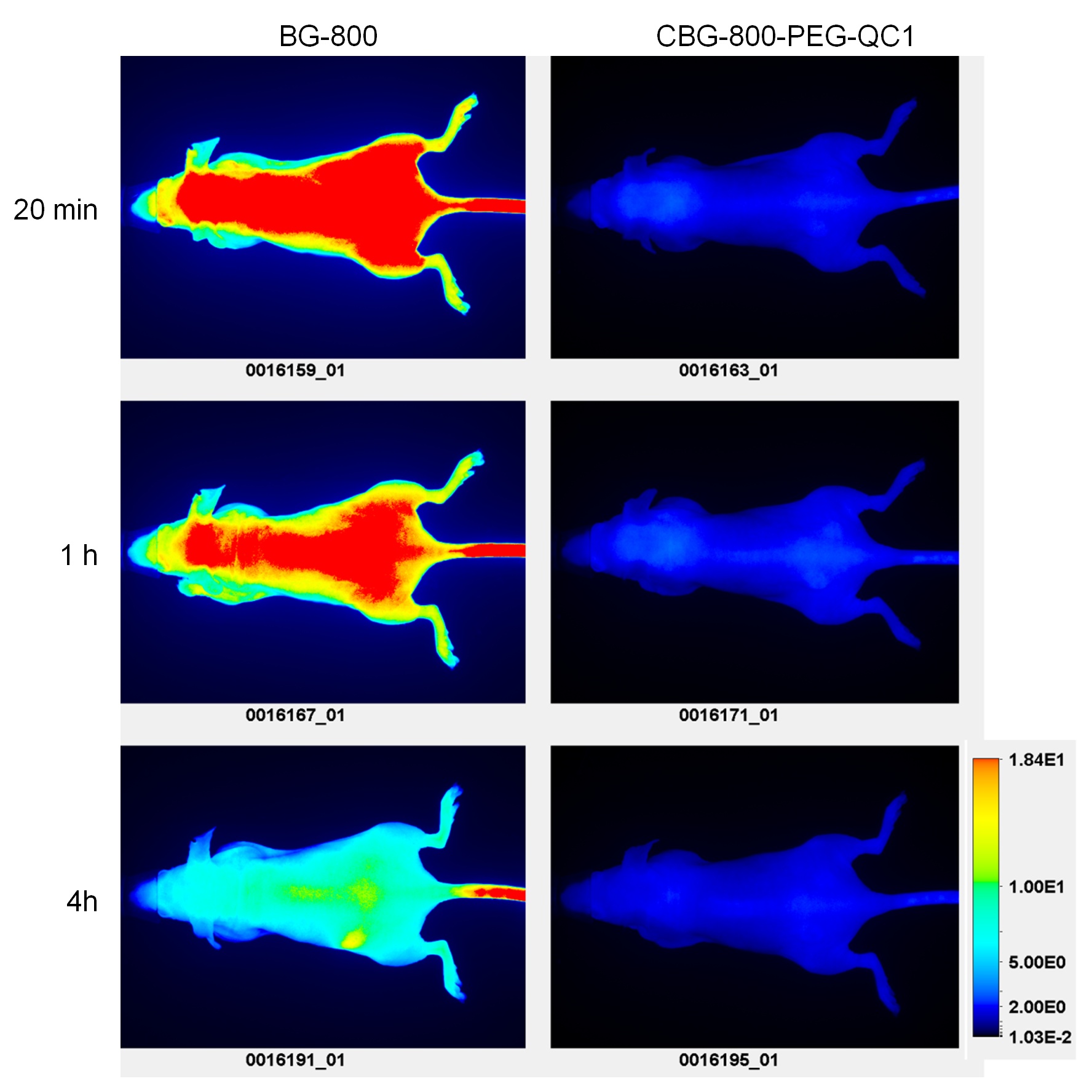

Supplement: Figure S2 — Comparison of BG-800 and CBG-800-PEG-QC1 in vivo . Nude mice were injected with 10 nmol BG-800 or CBG-800-PEG-QC1 and imaged at different time points. Note that the fluorescence signal of CBG-800-PEG-QC1-injected mouse was much lower than that of BG-800-injected mouse. (TIF) [file pone.0034003.s002.tif]
